# Case of Ascites

**Published:** 1863-02

**Authors:** J. Stearns

**Affiliations:** Pomeroy, Onondaga Co., N. Y.


### ART. III.—*Case of Ascites.*

[For the Buffalo Medical Journal.]

Mrs. Hubbard, aged fifty, of New Woodstock, N. Y., had, as I was informed, been dropsical a year or more. I operated and discharged from the abdomen, seventy-five pounds of water. She rather sunk after the first pailful, I stopped the discharge, gave stimulants and sustained the system well by occasionally taking time for the abdomen to collapse. I found some enlargment of the liver but no ovarian derangment.

J. STEARNS, M. D.,

Pomeroy, Onondaga Co., N. Y.

